# Analyzing the effects of pickling sludge and fly ash valorized cement sand bricks

**DOI:** 10.1038/s41598-025-08359-7

**Published:** 2025-07-04

**Authors:** Amit Bhomia, Srikanta Routroy, Anupam Singhal, Rahul Samyal

**Affiliations:** 1 Department of Mechanical Engineering, Birla Institute of Technology & Science, Pilani, Rajasthan 333031 India; 2 Department of Civil Engineering, Birla Institute of Technology & Science, Pilani, Rajasthan India

**Keywords:** Waste management, Performance analysis, Scanning Electron microscopy (SEM), Microstructural analysis, Stainless-steel pickling sludge, Composites, Mechanical properties, Civil engineering

## Abstract

The disposal of Stainless-Steel Pickling Sludge (SSPS) in landfills remains an important issue. Utilizing SSPS as construction material mitigates the negative environmental effects associated with its disposal, providing a sustainable solution. This study investigates co-utilization of SSPS and fly ash as partial substitution of river sand on cement sand bricks properties. Nine cement sand bricks compositions, including control mix, were prepared with varying composition of SSPS, fly ash and river sand. Four compositions were developed with SSPS varied from 2.5 to 10% with fixed fly ash content of 50%. Four additional compositions with varying fly ash content from 40 to 47.5% and varying SSPS 2.5–10% content as partial substitution of river sand were prepared. The developed bricks demonstrated that gradual increment of SSPS (2.5–10%) and reduction of fly ash (47.5–40%) proved incremental to the compressive strength up to 28 MPa. In addition, the morphological analysis using Scanning Electron Microscopy (SEM), X-ray Diffraction (XRD) and X-ray Fluorescence (XRF) were conducted for the compositions. The microstructure analysis showed that with inclusion of fly ash, Mix 2 (M2) compositions revealed a dense microstructure validating the sorptivity results as compared to Mix 1 (M1) compositions. Finally, the cost estimation of the waste valorized bricks as compared to the control bricks was observed to be significantly low. The experiment outcomes concluded adoption of SSPS-fly ash waste valorized bricks as a greener alternative to disposal.

## Introduction

The global stainless steel market has seen rapid growth over the years owing to the impressive corrosive resistance and physiochemical properties of steel^1^. These properties have led to the steel application in various sectors such as construction, transportation, industrial equipment, household appliances etc^2,3^. In stainless steel production, removal of oxide layers is essential for improving the surface appearance and corrosive resistance of steel. This is achieved through pickling process^4^. During pickling process, steel is immersed in acidic solutions such as H_2_SO_4_, H_3_PO_4_, HNO_3_ or HF to eliminate rust, dust and scale^5,6^. After pickling, this solution is neutralized with lime or other alkaline agents for harmless disposal. However, the neutralization process leads to the generation of pickling sludge, amounting to 3–5% of total stainless steel production^7^. In addition, disposal of this sludge waste in secured landfills poses severe environmental concerns^8^. Hence, sustainable waste management solution needs to be explored to deal with the environmental concerns associated with disposal of this waste.

1 $$\:2HF+{Ca\left(OH\right)}_{2}\:\to\:{CaF}_{2}+{2H}_{2}O$$

Waste valorization emerges as an innovative approach for waste management that involves transforming waste into valuable materials^9,10^. Recently, there has been a great interest in valorization of wide variety of waste in construction sector^11,12^. As valorizing of waste in construction sector reduces resource consumption and lowers the emissions^13^. Paving blocks or bricks are the most common building materials used in the construction sector. The large-scale production of these blocks requires significant amounts of non-renewable materials, such as river sand, leading to the depletion of natural resources^14,15^. In fact, the consumption of river sand in 2010 alone was 630 million tons, with an expected extraction requirement of 1.4 billion tons by 2020^16^. This overuse of river sand raises significant environmental concerns related to sand mining activities^17^. As a result, intensive investigations have been conducted incorporating various types of waste material such as recycled concrete waste^18,19^ crumb rubber^20,21^ glass wastes^22,23^ as partial or complete replacement of river sand in paving blocks or bricks. For instance, modified cement sand blocks were developed with peat soil partially replacing river sand. The blocks assessed for thermal conductivity, water absorption and compressive strength showed feasible results compared to the control mix^24^. Similarly, waste steel furnace slag partially replaced river sand in the paving block. The block with 40% slag content showed maximum compressive strength of 25 MPa^25^. Moreover, reclaimed asphalt pavement was used as a replacement for aggregates in concrete pavement blocks. The compressive strength of the blocks compared to the inclusion of fine aggregate revealed a significant reduction in compressive strength with the inclusion of coarse aggregates^26^. Utilization of these waste materials have provided a sustainable disposal alternative along with lower consumption of natural resources.

On the other hand, utilization of pickling sludge as a construction material has been of keen research interest, as shown in Table 1. For one thing, pickling sludge has depicted suitable characteristics for reuse in roadbed materials, concrete aggregate and bricks when stabilized with sodium sulphide hydrate^27^. In addition, pickling sludge utilized in clay bricks has shown lower Ni and Cr leaching values^28^. In a study, stainless steel pickling sludge and blast furnace bag dust with a metallization rate of 84.9% were used to develop de-sulfurized zinc and metallized pellets by reduction roasting method^29^. However, the increment of pickling sludge content in construction materials such as mortar and concrete has shown a decline in compressive strength^30^.

**Table 1:** Utilization of pickling sludge to develop different products.

Waste utilized	Value added product	References
Stainless steel pickling sludge and fly ash	Cement–sludge–fly ash concrete cubes	^31^
Municipal solid waste incinerated fly ash pickling sludge and glass waste	Parent glass and glass ceramic	^32^
Stainless steel pickling sludge	Cuspidine glass ceramic	^33^
Stainless pickling sludge	Parent glass and glass ceramics	^34^
Stainless steel pickling sludge	Low-carbon and low-sulfur Fe-Cr-Ni-Si alloy	^35^
Hot-dip galvanizing stainless steel pickling sludge	Fired clay hollow blocks, tiles and cubes.	^36^
Stainless steel sulfuric acid pickling bath sludge	Cement-sludge sand mortar cubes	^37^

To overcome this, supplementary cementitious materials such as glass-granulated blast furnace slag, silica fume, and fly ash are used to enhance the strength of cement-based products^38,39^. Among them, fly ash emerges as the most commonly used pozzolanic material. Due to its pozzolanic properties, fly ash, a waste by-product generated in coal thermal power plants, is used in cement-based products^40,41^. Fly ash with more than 70% content of (SiO_2_ + Al_2_O_3_ + Fe_2_O_3_) is defined as pozzolanic and is designated as Class ‘F’^42^. The silica in this fly ash reacts with Calcium Hydroxide (C-H) to form Calcium-Silicate-Hydrate (C-S-H), improving the mechanical strength of cement products^43^. For instance, mortar developed with 30% and 40% of fly ash as substitute of river sand showed higher compressive strength of 46.77 MPa and 34.93 MPa after 28 days of curing^44^. In addition, quarry waste as complete replacement of river sand along with 0 to 40% of fly ash in masonry blocks were used. The blocks with 40% fly ash as substitute of cement revealed improved thermal performance, higher compressive strength and acid resistance^45^.

From the review of the earlier studies, it is observed that the behavior of co-utilizing pickling sludge and fly ash in mortar paving bricks remains unexplored. Further, the detailed microstructural study based on the co-utilization of SSPS and fly ash as replacement of river sand on brick properties has not been explored. Hence, their combined effect on the paving brick properties warrants a thorough investigation.

Therefore, the work carried out in this study not only aims to divert waste material such as pickling sludge and fly ash from disposal to value added products but also focuses on reducing reliance on non-renewable resources such as river sand. Incorporation of these industrial waste in bricks can further help in mitigating the negative environmental impacts related to their disposal. In relation to this the main objectives of the current study are: (1) To analyze the effect of SSPS and fly ash as replacement of river sand on cement mortar brick properties. (2) To assess various properties of these bricks, such as compressive strength, water absorption, and microstructure characteristics. The outcomes of this study will provide valuable insights into the mechanical and microstructural properties of pickling sludge and fly-ash valorized bricks, thereby providing significant guidance for utilization in construction applications such as light-weight pedestrian paving bricks.

## Materials and methodology

### Materials

**Fig. 1:**
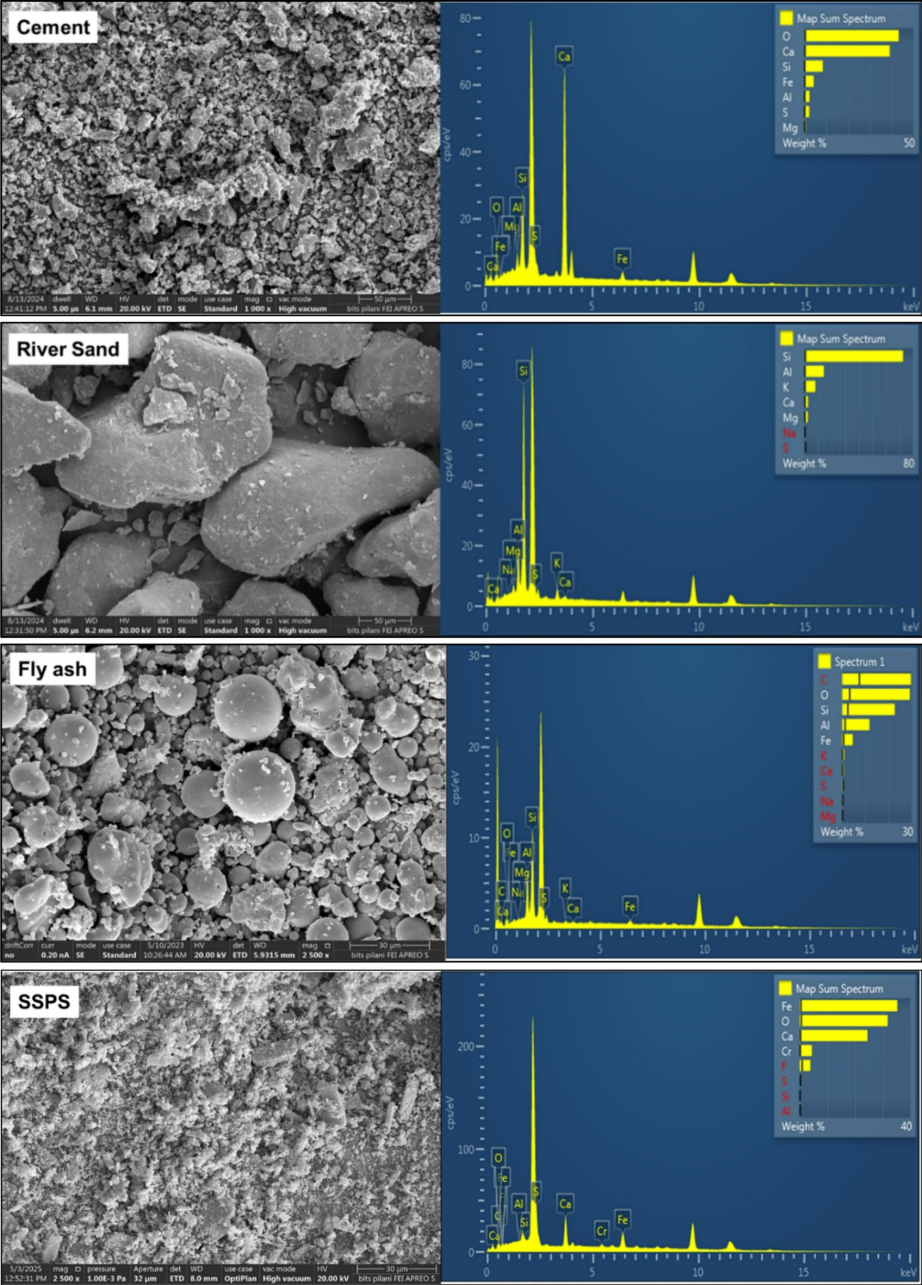
SEM-EDS result of cement, river sand, fly-ash and stainless steel pickling sludge (SSPS).

In this study, Ordinary Portland Cement (OPC) of 43-Grade is used as binder conforming to^46^. Nickle recovered stainless-steel pickling sludge sample was obtained from the JSW, Jharkhand, India. Fly ash was obtained from the National Thermal Power Corporation site in Jharli, Jhajjar district, India, conforming to (IS: 1727–1967). The preprocessing of the materials included drying for 24 h at 60 °C to remove moisture. The raw materials were further sieved through 150 μm. Dried sludge, cement, river sand and fly ash samples were investigated for their chemical composition using X-ray fluorescence (XRF) analysis with chemical characteristics depicted in Table 2. Figure 1 depicts the Scanning Electron Microscopy (SEM) and Energy Dispersive X-ray spectroscopy (EDS) analysis of cement, river sand, fly ash and pickling sludge. The SEM images show the irregular-shaped particles of the sludge and river sand. The SEM images, however, depict spherical and smooth particles of fly ash.

### Mix design and preparation

The composition of the mixes for brick preparation are denoted as Control Mix (CM), Mix 1 (M1) and Mix 2 (M2). These mixes are prepared using cement, river sand, pickling sludge, fly ash and water as the raw materials. The purpose of the mix design was to analyze the optimum fly ash and pickling sludge content in the bricks as a partial replacement of river sand. In the mixes, river sand content was reduced from 75 to 15%. M1 composition was designed to keep cement content constant at 25% and Fly ash content constant at 50%, whereas river sand was replaced by the SSPS to observe the effect of sludge on strength development. Further, M2 composition was designed to keep cement content constant at 25% and river sand content constant at 25%, whereas Fly ash was replaced by the SSPS to analyze the optimum achievable strength.

**Table 2:** XRF details of raw materials.

Oxides (%)	Cement	River sand	Fly ash	SSPS
Al_2_O_3_	4.536	14.22	29.73	-
SiO_2_	18.18	66.53	59.42	0.613
SO_3_	3.014	-	0.129	39.22
K_2_O	1.13	2.81	1.51	-
CaO	66.76	7.214	1.014	43.556
TiO_2_	0.54	1.18	2.03	-
Fe_2_O_3_	5.097	6.752	5.041	-
SrO	0.38	-	-	-
Ag_2_O	0.116	-	-	-
MnO	-	0.11	-	0.13
P_2_O_5_	-	0.73	0.69	-
Cr_2_O_3_	-	-	-	1.849
NiO	-	-	-	0.2
Loss on ignition (%)	0.247	0.454	0.436	0.128

#### Control mix

The composition of the control mix mainly contains Portland cement, river sand, and water. This control mix works as a baseline scenario for comparison with the modified composition incorporating SSPS and fly ash in varying range.

#### Mix 1 (M1)

In this composition of the bricks, four different bricks were prepared with 2.5, 5, 7.5 and 10% pickling sludge replacing river sand. The compositions are designated as S2.5F50, S5F50, S7.5F50 and S10F50. The quantities of pickling sludge and fly ash varied in these compositions, while the quantity of cement and fly ash was fixed.

#### Mix 2 (M2)

Four different bricks composition, S2.5F47.5, S5F45, S7.5F42.5 and S10F40, were prepared with 2.5, 5, 7.5 and 10% of pickling sludge replacing equal quantities of fly ash. In this mix, cement and river sand were kept constant while fly ash and pickling sludge content were varied.

**Fig. 2:**
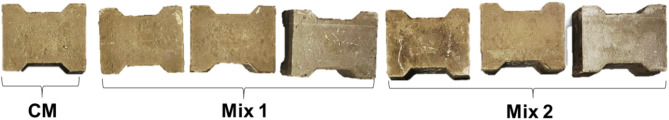
Different bricks composition.

The brick mixes were developed with various compositions, incorporating different proportions of pickling sludge (2.5–10%), fly ash (50 − 40%) and river sand (75 − 15%) by weight. The raw materials were first air-dried and subsequently mixed based on the compositions depicted in Table 3 using the dry mix fabrication method. The water-to-cement ratio for fabrication was 0.4. The mixed proportion of the raw materials was placed in a steel mould with the size of (200 mm x 160 mm x 82 mm), which was subsequently pressed at 3000 psi using a hydraulic machine for one minute. The mixture was de-moulded after the compaction. After the compaction, the bricks developed as illustrated in Fig. 2 were air-cured in a laboratory environment with 30 ± 5 °C and a relative humidity of 70 ± 5%.

**Table 3:** Compositions of cement sand bricks.

S.No	Mix notations	Extended mix notations	Raw material in dry state (% by wt.)			
			SSPS	Cement	River sand	Fly ash
1	Control mix	CM	0	25	75	0
2	M1	S2.5F50	2.5	25	22.5	50
3		S5F50	5	25	20	50
4		S7.5F50	7.5	25	17.5	50
5		S10F50	10	25	15	50
6	M2	S2.5F47.5	2.5	25	25	47.5
7		S5F45	5	25	25	45
8		S7.5F42.5	7.5	25	25	42.5
9		S10F40	10	25	25	40

Note: S- Sludge; F- Fly Ash, CM- Control Mix.

### Mechanical and durability testing

The mechanical properties of the developed cement sand bricks based on the compressive strength were calculated following Indian Standard Ordinary Portland Cement^47^. Moreover, durability property such as water absorption was also evaluated following Indian Standard Ordinary Portland Cement^47^. In water absorption, test brick samples, after the initial 28 days of curing, were dried in an oven at 105 °C for 24 h. After drying, the mass of the samples was cooled at room temperature and measured in the dry state (W_d_). Subsequently, the samples were immersed in water for 24 h and were wiped with a damp cloth to remove trace water from the surface to measure the wet mass of the bricks (W_w_). The percentage of water absorption for each sample was then calculated using Eq. 2.

2 $$\:Water\:Absorption\:\left(\%\right)=\:\frac{{\text{W}}_{\text{w}}-{\text{W}}_{\text{d}}}{{\text{W}}_{\text{d}}}\times\:100$$

Where, W_w_ is the wet mass and W_d_ is the dry mass of the sample.

### Microstructural analysis

The microstructure analysis of the mortar mixes, including SEM-EDS and XRD, was studied after a 28-day compressive strength test. The SEM analysis on the gold-plated brick sample was conducted using FEI Apreo LoVac. The analysis helped in evaluating the modification in the morphology of mortar mixes developed with the addition of pickling sludge and fly ash. Similarly, XRD analysis was performed using Bruker D2 Phaser with (2Θ range 10–60°, and scan rate of 2.5° per minute) on the brick samples.

## Results and discussion

This section discusses the various analyses through which the structural performance of the developed bricks was evaluated. The analysis begins with a mechanical and durability assessment of the developed bricks. For this, compressive strength based on varying compositions of stainless-steel pickling sludge and fly ash is evaluated. Moreover, the water absorption of the bricks was evaluated for durability purposes. Furthermore, microstructural analysis utilizing Scanning Electron Microscopy (SEM), Electron Dispersive Spectroscopy (EDS) and X-ray Diffraction (XRD) reveals details such as crystalline structures, unreacted particles, and micropores.

### Compressive strength

**Fig. 3:**
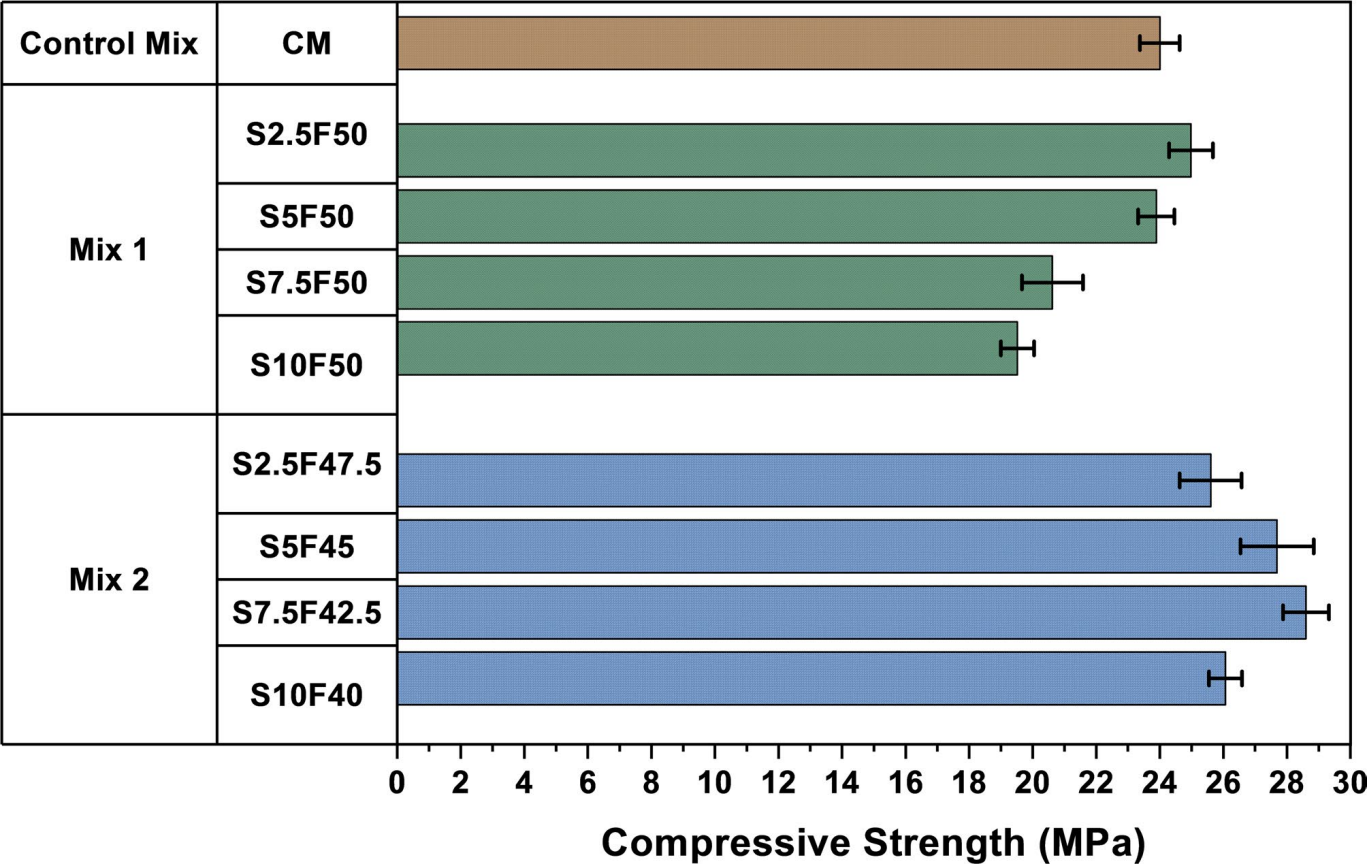
Compressive strength of different composition of bricks.

The compressive strength of the mixes was examined for their structural performance based on the varied fly ash and pickling sludge content as depicted in Fig. 3. The compressive strength of cement mortar Control Mix (CM) was observed as 24 MPa after 28 days of curing. Meanwhile, the Mix 1 (M1) compositions exhibit a clear trend where the compressive strength of the bricks declined as the percentage of stainless-steel pickling sludge was increased, with fly ash content kept constant. At 2.5% sludge, the average compressive strength is relatively high at 24.985 MPa, but as the sludge content increases to 5%, 7.5%, and 10%, the compressive strength progressively drops to 23.89 MPa, 20.625 MPa, and 19.525 MPa, respectively. This decline is attributed to the increment of sludge content along with gradual reduction of river sand. The increase in substitution amount leads to the dilution effect in the matrix leading to the reduction of the compressive strength^48^. In addition, this dilution effect introduces reduced binding efficiency and increased porosity, disrupting the matrix composition with reduction in compressive strength.

The decrease in compressive strength observed in the M1 compositions with percentage of stainless-steel pickling sludge increment is primarily due to several interrelated factors affecting the matrix. For instance, as the sludge content increases, the overall matrix composition is altered, with the sludge introducing components that may not effectively contribute to the structural integrity. This can lead to increased porosity and the formation of microstructural weaknesses, as the sludge particles compared to the supplementary material such as fly ash may not have enhanced packing density that contributes to compactness and strength increment^49^. Additionally, the chemical composition of the sludge could interfere with the cement hydration process, leading to incomplete hydration or the formation of non-beneficial phases, further compromising the strength. Together, factors such as reduced pozzolanic activity, disruption of the cementitious matrix, increased porosity, and potential chemical incompatibility result in a noticeable decline in compressive strength as the sludge content rises.

Further, for Mix 2 (M2) compositions, the compression test results of bricks demonstrated a noteworthy trend regarding the impact of varying proportions of stainless-steel pickling sludge and fly ash on the compression strength of the bricks. As the percentage of stainless-steel pickling sludge increases incrementally from 2.5 to 5%, 7.5% and finally to 10% of the total mixture, there is a consistent increase in compression strength. Moreover, decrement in fly ash quantity relates to the increment in strength till 42.5%. However, as fly ash contributes to the formation of a dense, strong matrix, and its reduction likely weakens the overall structure^50^. In addition, with further reduction of fly ash content by 2.5% compressive strength of the bricks gradually decreases.

Beginning with a mixture containing 2.5% stainless steel pickling sludge, the compression strength is recorded at 25.60 MPa. With a gradual increase in the proportion of sludge to 5%, the compression strength exhibits a notable improvement, reaching 27.69 MPa. Continuing this trend, as the sludge content is further increased to 7.5%, the compression strength rises to 28.605 MPa. However, on further increasing to 10% stainless steel pickling sludge content, the compression strength reduced to 26.07 MPa.

These results suggest that the incorporation of fly ash and stainless-steel pickling sludge into the brick mixture contributes positively to the mechanical properties, specifically enhancing compression strength. In addition, the M2 composition bricks fulfills the individual compressive strength requirement specified for non-traffic paving bricks incorporating waste^51^. The improvement in compressive strength of the bricks may be attributed to the pozzolanic activity of the fly ash, where CH from the cement and pickling sludge hydration reacts with reactive silica present in fly ash to form CSH. In pozzolanic reaction, negligible traces of unreactive fly ash particles illustrate complete reaction between the reactive silica and CH^52^. The improved strength makes bricks suitable for applications in construction where durability and load-bearing capacity are essential. Additionally, the developed bricks fulfill the requirement specified in standards depicted in Table 4.

**Table 4:** ASTM standards specified for bricks.

ASTM standard	Type	Compressive strength (Min.)	Water absorption (Max.)
C90-16a^53^	Lightweight	13.2 MPa	288 kg/m3
	Mediumweight	13.2 MPa	240 kg/m3
	Heavyweight	13.2 MPa	208 kg/m3
C902-15^54^	SX	55 MPa	8%
	MX	20.7 Mpa	14%
	NX	20.7 MPa	no limit

### Water absorption

**Fig. 4:**
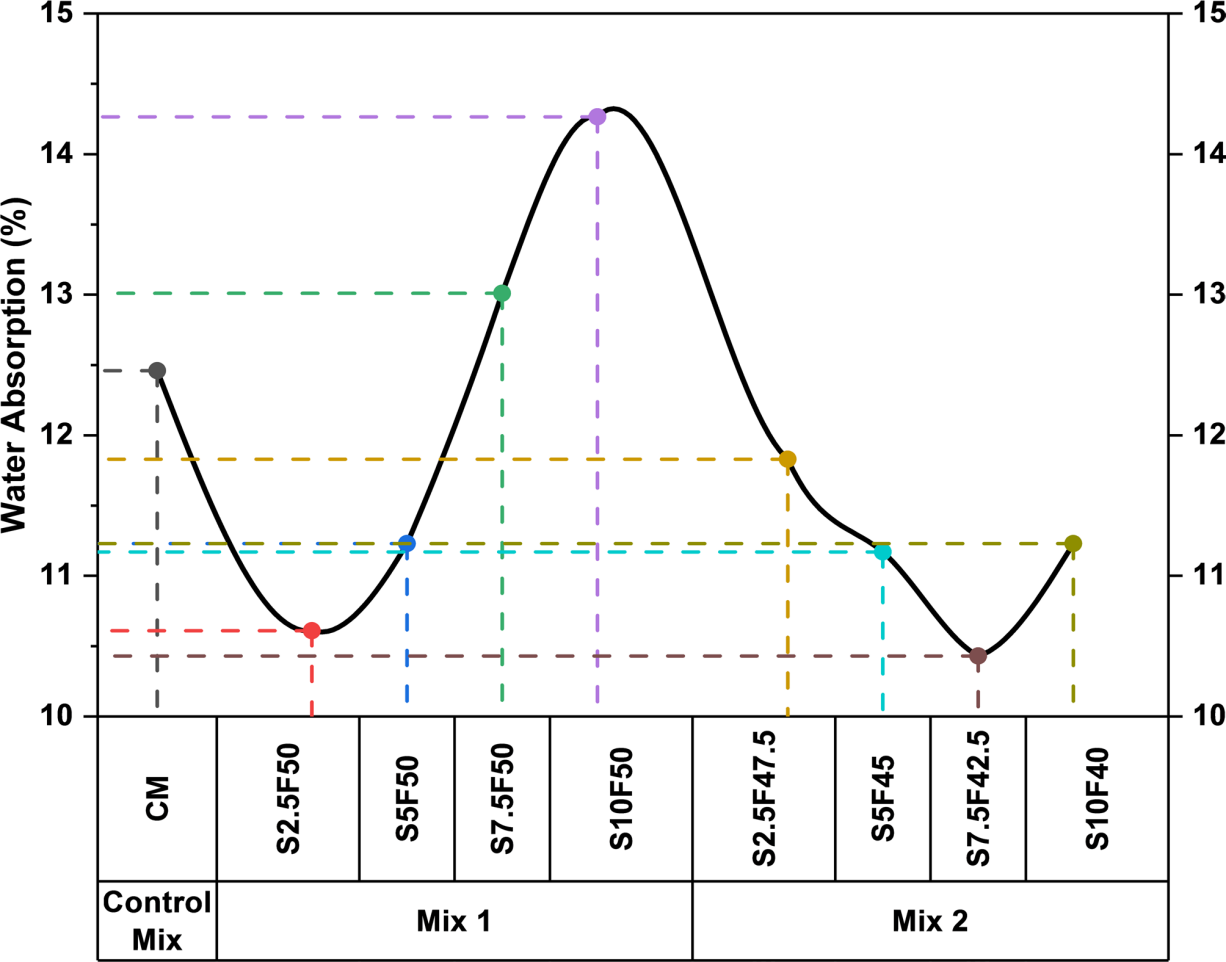
Water absorption of different brick compositions.

**Fig. 5:**
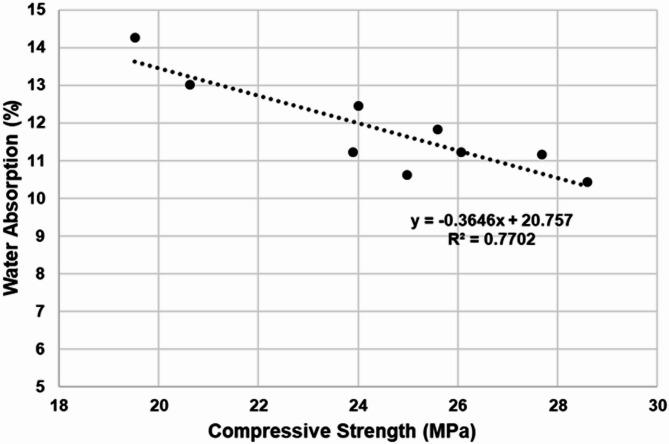
Correlation between water absorption and compressive strength.

Water absorption is performed to analyze the amount of water retained by different brick compositions under specified conditions. The results in Fig. 4 suggest average water absorption of different brick compositions. Beginning with the CM, water absorption of 12.46% was observed. Meanwhile, M1 compositions illustrate a clear trend when the brick matrix’s sludge and fly ash content were varied. It was observed that the content of sludge was increased from 2.5%. 5%, 7.5%, and 10%, the water absorption percentage of the mixes gradually increased from 10.61%, 11.23%, 13.00% and finally to 14.26%. The results of the water absorption test confirmed the water absorption requirement specified in Standard Specification for Loadbearing Concrete Masonry Units under ASTM Standard^53^ for the bricks. Further, M2 compositions, with increments in sludge content from 2.5%, 5%, and 7.5% decrement of 11.83%, 11.17% and 10.43% in absorption of water content was observed. The water absorption percentage declines due to the reduction in porous particles of the cement matrix with the inclusion of fly ash content^55^. However, an increment of 11.23% was observed, with a further increment of sludge content by 2.5% in the S10F40 mix. The increment in sludge content to 10% and reduction of fly ash content to 40% leads to formation of a porous structure, raising the water absorption in S10F40 matrix. Furthermore, the relationship between water absorption and compressive strength is depicted in Fig. 5. The correlation fitting in Fig. 5. illustrates an inverse relationship between the parameters. It showcases a linear reduction in compressive strength with increase in water absorption (%). Additionally, the graphical representation with (R^2^ value of 0.7702) reaffirms the relationship between the parameters.

### SEM analysis

**Fig. 6:**
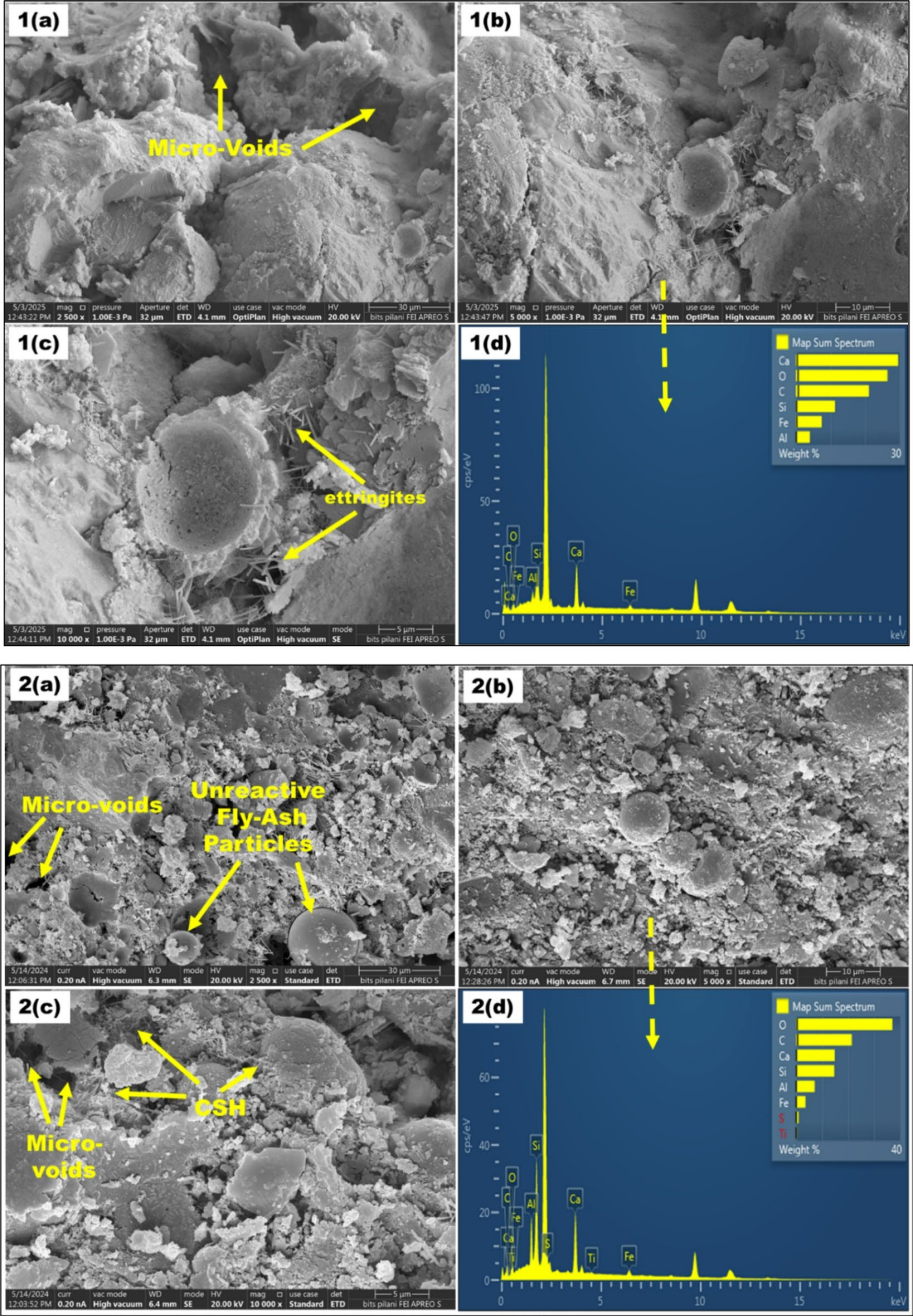

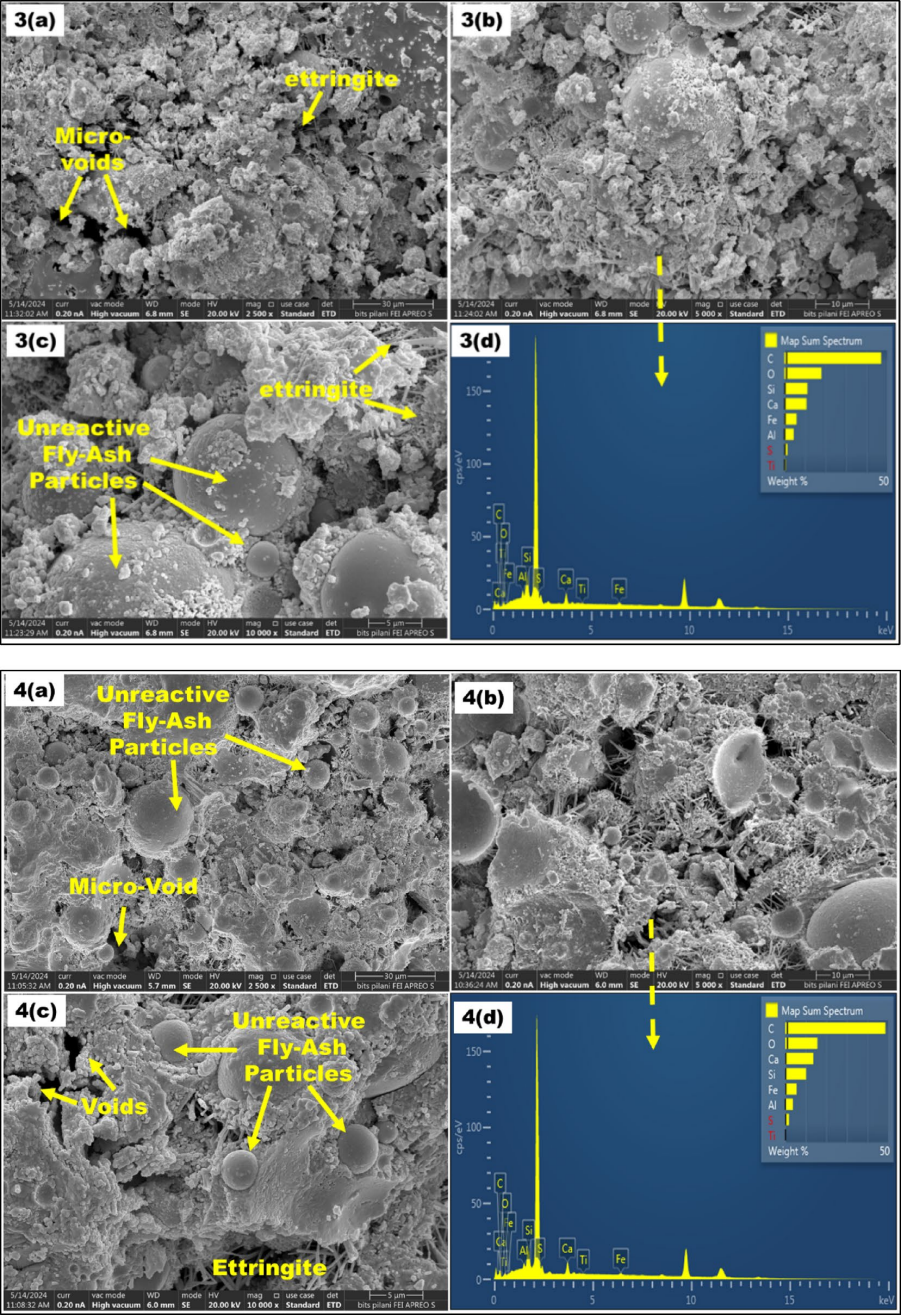

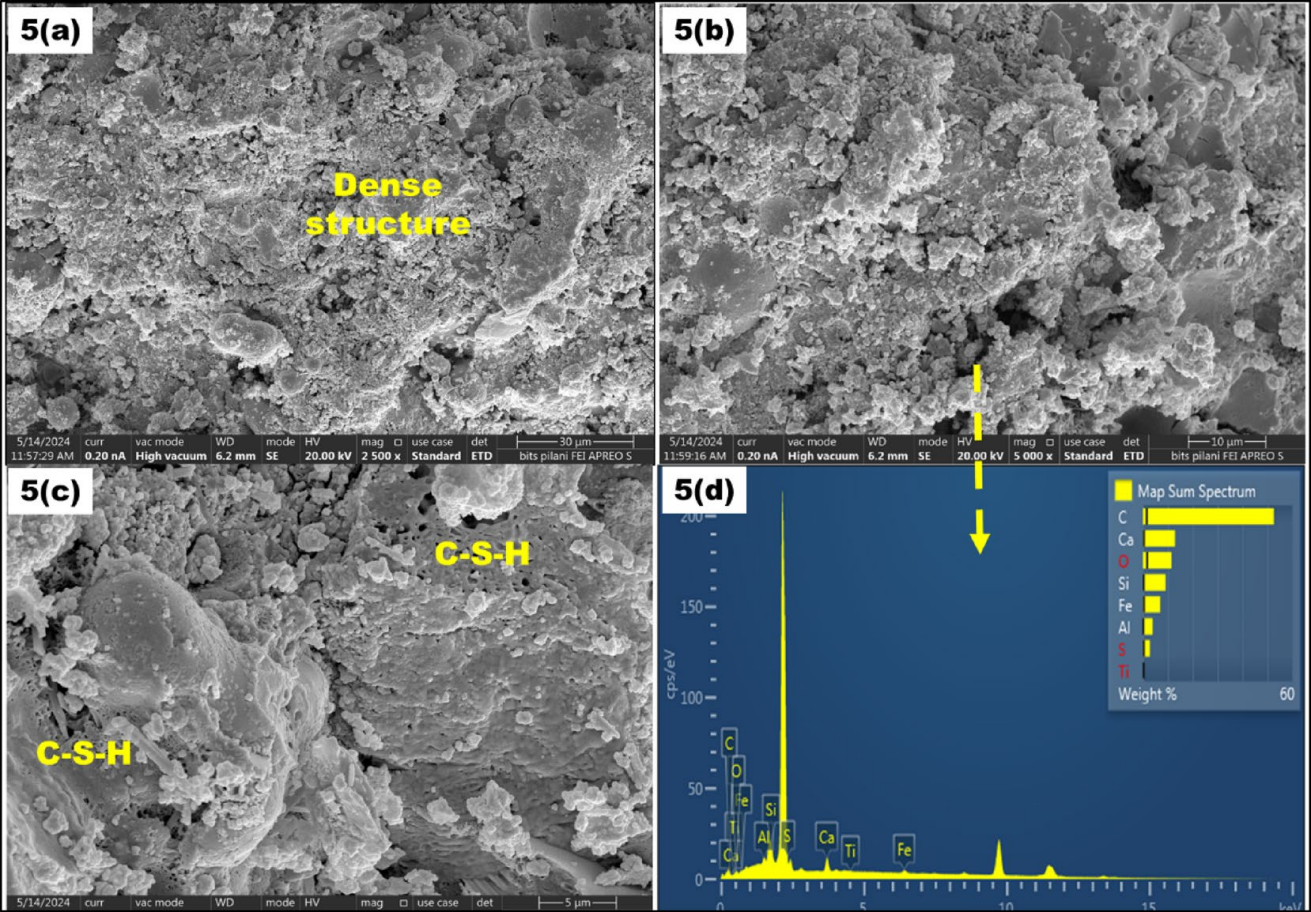
*SEM-EDS images (1–5) of CM*,* S2.5F50*,* and S10F50*,* S2.5F47.5*,* and S7.5F42.5 bricks*.

For SEM analysis, Control Mix (CM) with S2.5F50, S10F50, S2.5F47.5, and S7.5F42.5 were selected based on their highest and lowest compressive strength in the M1 and M2 compositions. In addition, SEM-EDS images for these mixes are illustrated in Fig. 6**(1–5)**. In SEM analysis, Calcium Silicate Hydrate (CSH), micro-voids and ettringite were the significant entities observed. The SEM-EDS analysis of CM shows structure with micro-voids and ettringite formation as evident from the Fig. 6**(1-a**,** b**,**c**,** d).** In addition, for the M1 compositions, with an increment of pickling sludge content from 2.5 to 10% and reduction of river sand content from 22.5 to 15%, there is a significant increase in pore area and voids. For instance, the S2.5F50 mix showed a denser matrix than the S10F50 mix, attributed to higher CSH formation as depicted in Fig. 6**(2-a**,** b**,**c)**, correlating for its highest compressive strength in the M1 compositions. The presence of hydration products such as CSH and calcium aluminum silicate hydrate (CASH) phases contributes significantly to the matrix formation and strength enhancement^56^. Meanwhile, incrementing sludge content to 10% and reduction of river sand to 15% in composition S10F50 contributed to a porous structure with insufficient hydration products owing to the dilution effect^57^. The increased voids and excessive unreactive fly ash content in the composition can be observed in Fig. 6**(3-a**,** b**,**c)**, correlating to the declined strength^58^. However, for M2 composition, Fig. 6**(4-a**,** b**,**c)** and Fig. 6**(5-a**,** b**,**c)** substantiate that with an increment in sludge content (2.5–7.5%) and reduction in fly ash content (47.5–42.5%) a higher compressive strength due to the formation of CSH accompanying pore reduction is observed. The strength increment can be attributed to the pozzolanic reaction between silica present in fly ash and CH formed during hydration of lime present in cement and pickling sludge to produce hydration products^59^. Meanwhile, this rection of fly ash and calcium hydration product formed from lime present in cement and sludge shows synergy between the blend. Moreover, the dense structure of S7.5F42.5 in Fig. 6**(5-a**,** b**,**c)** further confirms the dissolution of fly ash particles compared to the S2.5F47.5 composition validating the highest compressive strength in the brick composition. Further, the outcomes of the SEM analysis aligned well with the compressive strength test conducted in this study.

### XRD

**Fig. 7:**
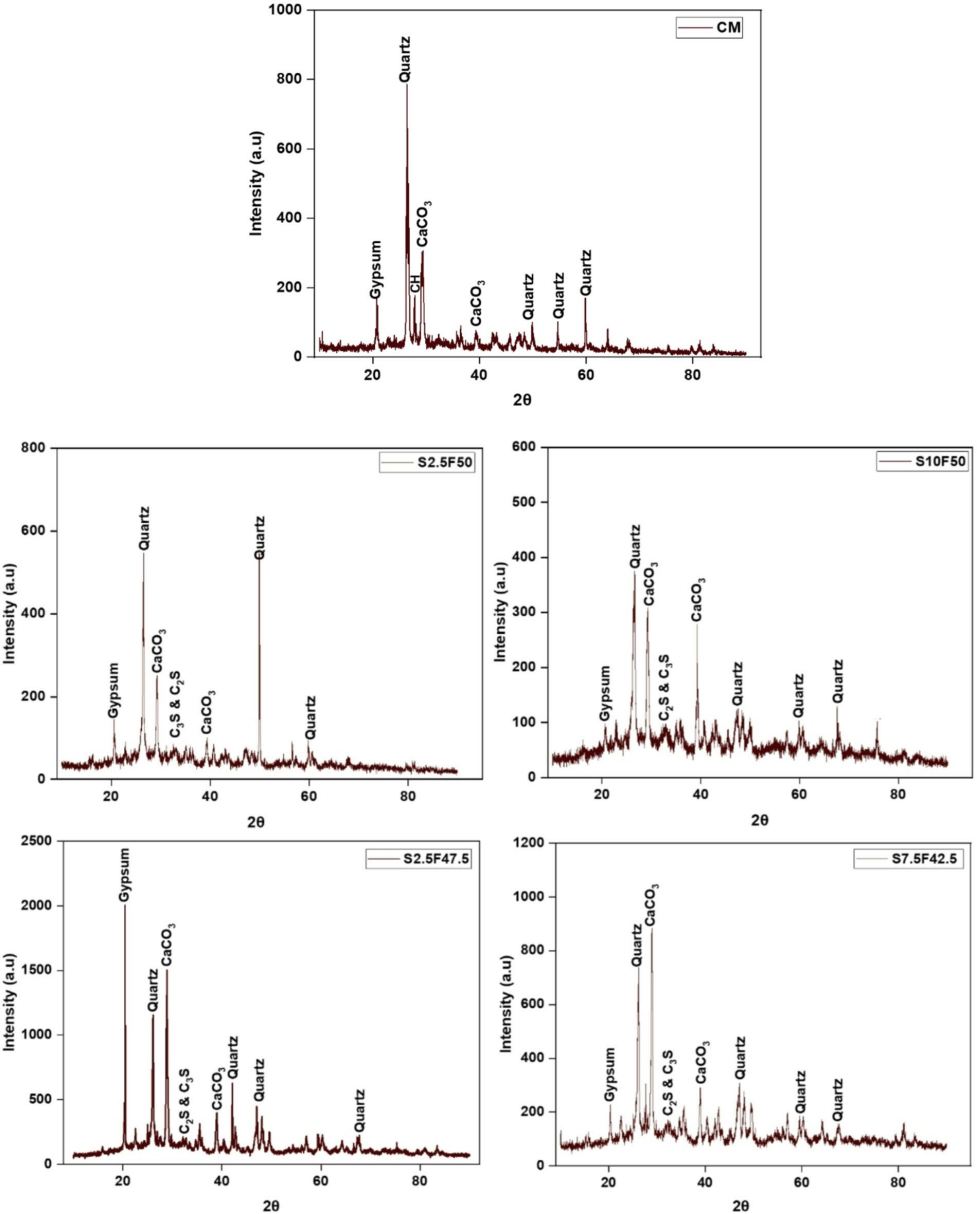
XRD analysis of CM, S2.5F50, S10F50, S2.5F47.5 and S7.5F42.5 compositions.

For XRD analysis, control mix CM, S2.5F50, S10F50, S2.5F47.5, and S7.5F42.5 mixes were selected based on the highest and lowest compressive strength from the M1 and M2 compositions. From the analysis, significant compounds, such as quartz, silicon dioxide, and calcium carbonate, with few traces of calcium silicates were observed. The increment in sludge content in each composition can be attributed to calcium content observed in the mixes. In Fig. 7, the XRD analysis for the selected matrix showed Quartz (SiO_2_) peak appears at 2θ = 26.6° in each composition as observed by Chen et al.^60^. In addition, the diffraction peak of calcium carbonate appears at 2θ = 29.5° and 39.5°, and peaks of calcium silicates (C_2_S and C_3_S) occurred at 2θ = 32.4° in M1 and M2 compositions. In M1 compositions, when sludge content increases from 2.5 to 10%, a higher peak intensity of calcium carbonate was observed in the S10F50 mix compared to the S2.5F50 mix. The gradual increment in sludge content increases the substitution content leading to a more pronounced dilution effect in M1 composition matrix^61^. However, for the M2 compositions, with the addition of sludge content from 2.5 to 7.5%, an increased intensity of calcium carbonate products was observed in the S2.5F47.5 mix compared to the S7.5F42.5 mix. The excessive amount of calcium carbonate in the mix S2.5F47.5 leads to the reduction in compressive strength of the mix by hindering the hydration process^62^.

### Cost stimation of developed bricks

The developed bricks incorporating pickling sludge and fly ash provide mechanical properties similar to CM. However, it becomes prudent to evaluate different compositions of bricks based on their economic feasibility. The cost calculation for different brick compositions is calculated in rupees (Rs) based on the requirement of raw materials quantity for covering a one-meter square area by the developed bricks, as depicted in Table 5. The cost calculation for different brick compositions is carried out based on the current rates of raw materials followed in Hisar district, Haryana, India. Further, the transportation distance of SSPS and fly ash are considered 10 km and 100 km, respectively. However, other raw materials such as river sand, cement and water are considered to be locally available.

**Table 5:** Unit cost of raw materials for 1m2 area covered by different composition of bricks.

Mix notations	Cement	River sand	Fly-ash (transportation cost)	SSPS (transportation cost)	Total cost of 1m^2^ area covered by bricks
CM	249.9	62.118	0	0	312.018
S2.5F50	239.19	16.065	42.84	0.714	298.809
S5F50	239.19	14.28	42.84	1.428	297.738
S7.5F50	239.19	12.495	42.84	2.142	296.667
S10F50	239.19	10.71	42.84	2.856	295.596
S2.5F47.5	249.9	20.706	33.915	0.714	305.235
S5F45	249.9	20.706	32.13	1.428	304.164
S7.5F42.5	249.9	20.706	30.345	2.142	303.093
S10F40	249.9	20.706	28.56	2.856	302.022

*S- Sludge; F- Fly Ash*,* CM- Control Mix*.

**Fig. 8:**
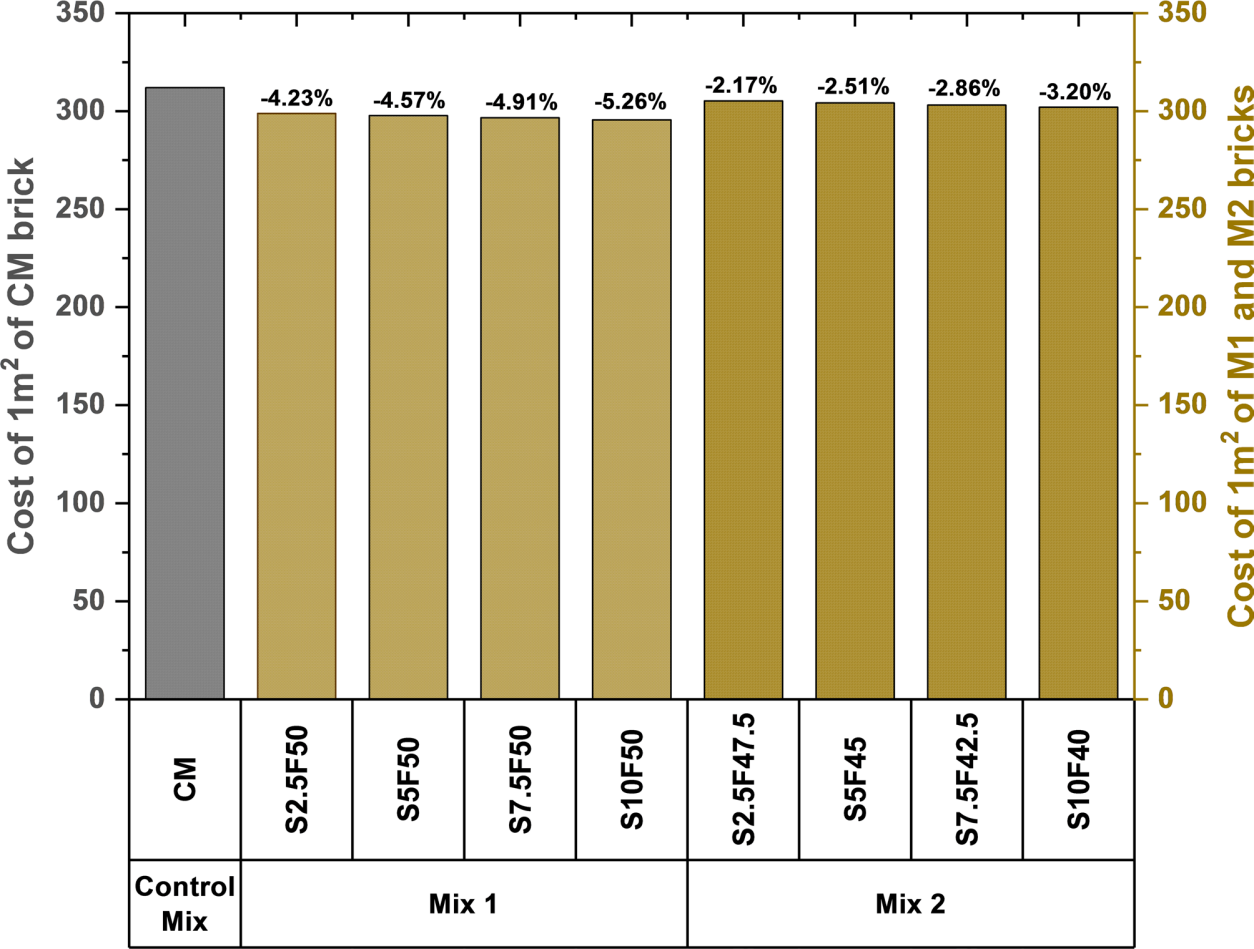
Cost analysis of different bricks compositions.

The cost analysis of the bricks developed with the inclusion of fly ash and SSPS showed a reduction in the cost compared to the control mix CM. The cost difference in developed bricks relative to the control mix is illustrated in Fig. 8. The production of M1 bricks showed significant reductions of 4.23, 4.57, 4.91 and 5.26% for S2.5F50, S5F50, S7.5F50 and S10F50 compared to the control mix. In addition, for M2 bricks, the cost reduction for S2.5F47.5, S5F45, S7.5F42.5 and S10F40 composition was 2.17, 2.51, 2.86 and 3.20% in relation to the control mix composition. The cost estimation results revealed the economic feasibility of pickling sludge bricks compared to the control mix. The outcomes of the cost analysis substantiate the economic feasibility of the SSPS and fly ash valorized bricks. The waste valorized bricks are not only economical as compared to the control mix but provides significant reduction in use of natural resources such as river sand.

## Conclusion

This study aimed to explore the potential possibility of co-utilization of Stainless steel pickling sludge (SSPS) and fly ash into cement sand bricks as replacement for sand. The outcomes of the tests concluded the feasibility of using SSPS and fly ash as replacement material in mortar based compositions. Moreover, from the analysis following conclusions are observed:

1. The increasing content of SSPS was detrimental to water absorption in bricks. For Mix 1 (M1) composition, continuous increment in water absorption was observed with increment of sludge content (2.5–10%). However, For Mix 2 (M2) compositions, with increment of SSPS (2.5–7.5%) and reduction of fly ash (47.5–42.5%) showed reduction in water absorption.
2. The compressive strength of the bricks decreased significantly in M1 compositions when SSPS content was increased (5–10%) due to the increased voids and excessive unreactive fly ash content in the composition. However, the bricks exhibited a rise in 28-day compressive strength with increment of SSPS content till 7.5% in M2 compositions, owing to the pozzolanic reaction, which got reduced with further increment of sludge content to 2.5%.
3. In SEM analysis, porous structure of bricks was observed with gradual increment of SSPS content in M1 compositions. However, M2 compositions revealed a denser matrix with increment in SSPS content till 7.5% and reduction of fly ash (47.5–42.5%).
4. The XRD analysis performed on the bricks, revealed higher calcium carbonate formation for each composition due to the lime content of cement and pickling sludge utilized in the brick compositions.
5. The cost estimation revealed significant reduction in cost for M1 and M2 compositions. The highest reduction in cost with 5.26% was observed for S10F50 compared to the control mix cost.

The results of the current research investigation revealed effects of SSPS and fly ash co-utilization in cement sand brick properties. It can be deduced from the results that adoption of pickling sludge and fly ash incorporated bricks can help in reduction of river sand without affecting the mechanical integrity of the aforementioned bricks. The incorporation of hazardous waste such as pickling sludge and fly ash in construction materials such as bricks can prove beneficial to the environment. The incorporation of these waste in bricks avoids landfilling based impacts and cost to the environment providing a sustainable solution.

## Data Availability

This manuscript has no associated data; all the data is disclosed within the manuscript.
